# The Specificity of Targeted Vaccines for APC Surface Molecules Influences the Immune Response Phenotype

**DOI:** 10.1371/journal.pone.0080008

**Published:** 2013-11-11

**Authors:** Gunnveig Grødeland, Siri Mjaaland, Gro Tunheim, Agnete B. Fredriksen, Bjarne Bogen

**Affiliations:** 1 Institute of Immunology, University of Oslo and Oslo University Hospital, Oslo, Norway; 2 K.G. Jebsen Centre for Influenza Vaccine Research, University of Oslo, Oslo, Norway; 3 Centre for Immune Regulation (CIR), University of Oslo and Oslo University Hospital, Oslo, Norway; 4 Division for Infectious Disease Control, Department of Bacteriology and Infection Immunology, Norwegian Institute of Public Health, Oslo, Norway; University of Delhi, India

## Abstract

Different diseases require different immune responses for efficient protection. Thus, prophylactic vaccines should prime the immune system for the particular type of response needed for protection against a given infectious agent. We have here tested fusion DNA vaccines which encode proteins that bivalently target influenza hemagglutinins (HA) to different surface molecules on antigen presenting cells (APC). We demonstrate that targeting to MHC class II molecules predominantly induced an antibody/Th2 response, whereas targeting to CCR1/3/5 predominantly induced a CD8^+^/Th1 T cell response. With respect to antibodies, the polarizing effect was even more pronounced upon intramuscular (i.m) delivery as compared to intradermal (i.d.) vaccination. Despite these differences in induced immune responses, both vaccines protected against a viral challenge with influenza H1N1. Substitution of HA with ovalbumin (OVA) demonstrated that polarization of immune responses, as a consequence of APC targeting specificity, could be extended to other antigens. Taken together, the results demonstrate that vaccination can be tailor-made to induce a particular phenotype of adaptive immune responses by specifically targeting different surface molecules on APCs.

## Introduction

The introduction of mass vaccination represents a major breakthrough for modern medicine. Thus far, most vaccines have been developed empirically, with the most successful vaccines being attenuated pathogens mimicking a natural infection[1]. Attenuated vaccines generally induce strong antibody and T cell responses, and a single immunization is often sufficient for obtaining life-long protection. However, live vaccines raise several safety concerns, and alternatives such as inactivated pathogens or subunit vaccines are often used instead, despite their reduced immunogenicity.

The effect of subunit vaccines can be increased by adding adjuvants to vaccine formulations, thereby influencing the magnitude and phenotype of immune responses. Vaccine formulations with alum, for example, tend to induce Th2 responses[2], characterized by CD4^+^ T cells secreting interleukin-4 (IL-4), IL-5, IL-9 and IL-13 and expression of the transcription factor GATA-binding protein 3 (GATA-3)[3]. Th2 cells help B cells[4], and mediate immunoglobulin (Ig) class swiching to IgG1 in mice[5]–[7]. Vaccine formulations with the adjuvant monophosphoryl lipid A (MPL), on the other hand, preferentially induce a Th1-like immune response[8], characterized by CD4^+^ T cells secreting the hallmark cytokine interferon γ (IFNγ), expression of the transcription factor T-bet[9], and Ig class switching to IgG2a[7].

Immunogenicity of subunit antigens may also be increased by targeting of antigen to antigen presenting cells (APCs). Such targeting may be achieved by coupling of antigen to APC-specific antibodies either chemically[10]–[13] or genetically[14]–[26]. For genetically constructed vaccines, antigens may be targeted by use of APC-specific complete Ig[15], [16], [24], APC-specific scFv[20], [23], or APC-specific natural ligands such as TLR ligands or chemokines[17], [22], [25], with antigen attached C-terminally.

An interesting issue is whether the specificity of the APC-targeted vaccine molecule can influence the phenotype of immune responses. In this respect, it has been shown that targeting of OVA to different subsets of dendritic cells (DCs) preferentially induce CD4^+^ or CD8^+^ T cells[24], but it is unclear whether this effect is due to the specificity for particular surface molecules, or to the surface molecules being expressed on a particular APC. Furthermore, fusion vaccines consisting of chemokines and antigens have been demonstrated to efficiently cross-present antigens on MHC class I molecules[21], [22]. Efficient activation of Th1 type CD4^+^ cells and cytotoxic T lymphocytes (CTL) has also been demonstrated following targeting to TLR7/8[19]. Improved humoral immunity has been demonstrated following targeting of vaccines to TLR5[26], and antigen fused to CTLA4 has been shown to increase IgG1 responses[15]. The mechanisms behind efficient induction of either cellular or humoral immunity, or both, have yet to be elucidated.

We have previously developed Ig-based homodimeric fusion vaccine proteins where each monomer consists of a targeting unit, a dimerization unit and an idiotypic (Id) scFv antigenic unit from malignant B cells[20]. Targeting of such vaccine molecules to MHC class II molecules[20], CD40[23] and chemokine receptors[22], [25] increased protective anti-Id immune responses against myelomas and B cell lymphomas. However, it has not been tested whether the different APC-specificities of the targeting units induce different types of immune responses. To investigate this, we have here compared two different targeting units (anti-MHC II and MIP-1α) for their ability to induce protective B and T cell responses against influenza hemagglutinin (HA). We demonstrate that while MHC class II targeting primarily induces antibody/Th2 immunity to HA, targeting to chemokine receptors predominantly results in CD8^+^/Th1 cell mediated immunity. The observed polarization is extendable to other antigens, as the same trends were observed when vaccinating with targeted OVA antigen. To our knowledge, the APC-receptor dependent immune polarization to Th1 or Th2 has previously not been investigated. The observed differences in elicited immune phenotypes can be exploited to construct vaccines tailor-made for inducing the desired immune response against a given pathogen.

## Materials and Methods

### Cloning of vaccine constructs

Vaccine molecules were constructed by inserting HA (aa 18–541) from influenza A/PR/8/34 (H1N1) or ovalbumin (OVA) into the cloning sites of the previously described pLNOH2 CMV-based vector[20], [22], [27]. HA was picked up from the plasmid HAwt-pCMV (kind gift from Harald von Boehmer) by primers that had been designed with fixed restriction sites for SfiI on the 5′ and 3′ ends: HA_18_5′; gag gcc tcg gtg gcc tgg aca caa tat gta tag gct acc and HA_541_3′: gga tcc ggc cct gca ggc ctc aca gtg aac tgg cga cag. The OVA gene was bought from GenScript with flanking SfiI sites. A vector encoding only HA (aa 18–541) was prepared by first mutating internal HA BsmI sites (silent mutations), and then moving the construct into the pLNOH2 vector (primer with fixed restriction site for BsmI in the 5′ end: ggt gtg cat tcg aca caa tat gta tag gct acc a, and the 3′ end primer described above)[27].

### Characterization of fusion vaccine proteins

DNA plasmids encoding the different vaccine proteins were transfected into HEK293E cells, as previously described[27]. Prior to assaying, the harvested supernatants were centrifuged at 13 000 rpm for 4 min. For Western blotting, vaccine proteins were run on a Novex 4–12% Tris-Glycine gel (Invitrogen) together with a SeeBlue Plus2 Prestained Standard (LC5925, Invitrogen), blotted (Immun-Blot PVDF membrane, 162–0177, BioRad) and incubated with a biotinylated anti-HA antibody (H36-4-52, kind gift from Siegfried Weiss)[28] and Streptavidin-HRP (RPN1231V, GE Healthcare), or anti-OVA (ab17293, Abcam) and anti-mouse IgG-HRP (1030-05, Southern Biotech). The membrane was developed with the ECL Western Blotting analysis system (RPN2109, GE Healthcare) and analysed on a Kodak Image station 200R with Molecular Imaging Software v 4.0.5.

Harvested supernatants were also analysed in triplicates in Sandwich ELISAs using 2 µg/ml of mouse anti-human IgG (C_H_3 domain) mAb MCA878G (AbD Serotec) as coat. Detection was performed with 1 µg/ml of biotinylated anti-HA mAb or biotinylated anti-human IgG (B3773, Sigma) and Strep-alkaline phosphatase (GE Healthcare). Plates were developed using Phosphatase substrate (P4744-10G, Sigma Aldrich) dissolved in substrate buffer, and read with a Tecan reader using the Magellan v5.03 program.

The chemotactic integrity of MIP-1α-HA and MIP-1α(C11S)-HA was assessed *in vitro*, as previously described[25], by quantifying Esb/MP cell migration across a 5 µm pore polycarbonate membrane in response to the titrated presence of vaccine proteins or a positive control (recombinant LD78β, Peprotech). Results from duplicate samples (mean) are presented as chemotactic index, defined as the fold increase of cells migrating in the presence of chemotactic factors over the spontaneous cell migration (i.e. in the presence of medium alone).

### FACS

MHCII I-E^d^-transfected L cell fibroblasts (CA36.2.1) were FcγR-blocked by incubation with 30% heat aggregated rat serum and 0,1 mg/ml 2.4G2 mAb, and then successively stained with affinity-purified vaccine proteins (10 µg/ml), biotinylated anti-HA antibody (H36-4-52, 1 µg/ml) and Streptavidin-PE (1 µg/ml) (554061, PharMingen). Cells were fixed with 2% paraformaldehyde, and analysed on a FACS Calibur flow cytometer (BD biosciences). Splenocytes from BALB/c mice were blocked and stained with vaccine proteins as above, but here the staining solution also contained FITC-conjugated anti-CD3 (1535-02, Southern Biotech) and APC-conjugated anti-CD11b (550993, BD Pharmingen) antibodies. Splenocytes were analysed on a LSRI flow cytometer (Becton Dickinson), and data analysed with the FlowJo software (Version 7.6).

### Detection of serum anti-HA or anti-OVA antibodies

Sandwich ELISAs were performed with either inactivated A/PR/8/34 (H1N1) (PR8) virus (Charles River) (1∶1600 in PBS) or OVA protein (A5503, Sigma) (2 µg/ml) as coat, and detected with biotinylated anti-IgG (A2429, Sigma Aldrich), anti-IgG1^a^ (553599, BD Pharmingen), anti-IgG2a^a^ (553502, BD Pharmingen), anti-IgG2b (553393, BD Pharmingen) or anti-IgG3 (406803, BioLegend), as previously described[27]. Hemagglutination-inhibition (HI) and micro neutralization assays were performed with PR8 as previously described[27].

### Viruses

A/Puerto Rico/8/34 (H1N1) (PR8) (kind gift from Dr. Anna Germundsson, National Veterinary Institute, Norway), was propagated by inoculating virus into the allantoic cavity of 10-day-old embryonated chicken eggs. TCID_50_ in pooled allantoic fluid was determined.

### Mice

Six to eight week old female BALB/c mice (Taconic, Denmark) were used. Animals were housed under minimal disease conditions. All animal experiments were approved by the National Committee for Animal Experiments (Oslo, Norway).

### Vaccination and viral challenge

Mice (n = 6/group) were anaesthetized (Hypnorm/Dormicum: 0,05 ml working solution per 10g s.c.) and shaved in the lower back region. Twentyfive µl of plasmids (purified from Endofree Qiagen kit (Qiagen)) dissolved in NaCl (a total of 25 µg DNA), were injected intradermally on each flank of the mouse, immediately followed by skin electroporation (EP) with DermaVax (Cellectis).

For viral challenge, anaesthetized mice received 5xLD_50_ of PR8 in 10 µl per nostril, as previously described[27]. Following viral challenge, mice were monitored for weight loss. The endpoint was a 20% weight reduction, as decided by the National Committee for Animal Experiments.

### Quantitative PCR

Quantitative RT-PCR was performed as previously described[27]. Briefly, mice were nasally flushed with 1 ml PBS/BSA (2%), and RNA extracted from 250 µl of the nasal wash using NucliSens® easyMagTM (Biomèrieux). Quantitative RT-PCR was performed with samples in triplicates, and with the following program using a Stratagene RealTime machine (Qiagen OneStep RT-PCR kit): 50°C (30 min), 95°C (2 min), followed by 45 cycles: 95°C (15 sec), 55°C (30 sec).

### ELISpot assay

ELISpot assays were performed as previously described[27]. Briefly, multiscreen HTS plates (Millipore) were coated with 12 µg/ml anti-mouse IFNγ (AN18)[29] or anti-mouse IL-4(11B11). Following blocking, single cell suspensions were prepared individually from spleens of vaccinated mice (n = 6/group), and stimulated with either class II restricted HA peptides (HNTNGVTAACSHEG or SVSSFERFEIFPK), a class I restricted HA peptide (IYSTVASSL) (0.8 µg/ml) (ProImmune), a control peptide (GYKDGNEYI), OVA (Sigma) or inactivated PR8 (Charles River). IFNγ or IL-4 producing cells were detected by biotinylated anti-mouse IFNγ (1 µg/ml) (XMG1.2, Pharmingen) or biotinylated anti-mouse IL-4 (1 µg/ml) (554390 BD Pharmingen) and Streptavidine alkaline phosphatase (1∶3000) (GE Healthcare).

### Interferon-γ ELISA

Single cell suspensions were prepared from spleens of vaccinated mice (n = 6/group), and stimulated with either class II restricted HA peptides (HNTNGVTAACSHEG and SVSSFERFEIFPK, 1∶1), a class I restricted HA peptide (IYSTVASSL) (ProImmune), inactivated PR8 (Charles River) (2 µg/ml) or medium alone. Supernatants were examined in Sandwich ELISAs with anti-IFNγ mAb (AN18) as coat, and with biotinylated anti-IFNγ (XMG1.2, Pharmingen) for detection. A standard curve of diluted and purified IFNγ was used to assess the concentration of IFNγ in sera.

### T cell depletion

Mice were DNA/EP vaccinated once with 25 µg αMHCII-HA, MIP-1α-HA or NaCl (n = 6/group). From day 12 and until termination of the experiment, groups of mice vaccinated with MIP-1α-HA were injected every other day i.p. with 400 µg of either purified anti-CD4 (GK1.5, ATCC)[30], or anti-CD8 (TIB105, ATCC)[31], or both, or control mAbs (SRF8-B6 and Y13-238). The mice vaccinated with αMHCII-HA were, every other day from day 12, injected i.p. with 400 µg of anti-CD4 and anti-CD8. On day 14, mice were challenged with PR8 and monitored for weight loss.

### Statistical analyses

Statistical analyses of antibody responses in sera were performed using one way Anova and Bonferroni's multiple comparison test with the Graphpad Prism software (GraphPad Software Inc. version 5). All other analyses were performed using the nonparametric Mann-Whitney test (one-tailed value) with Graphpad Prism software.

## Results

### Fusion gene cloning and functional characterization of vaccine fusion proteins

**Figure 1:**
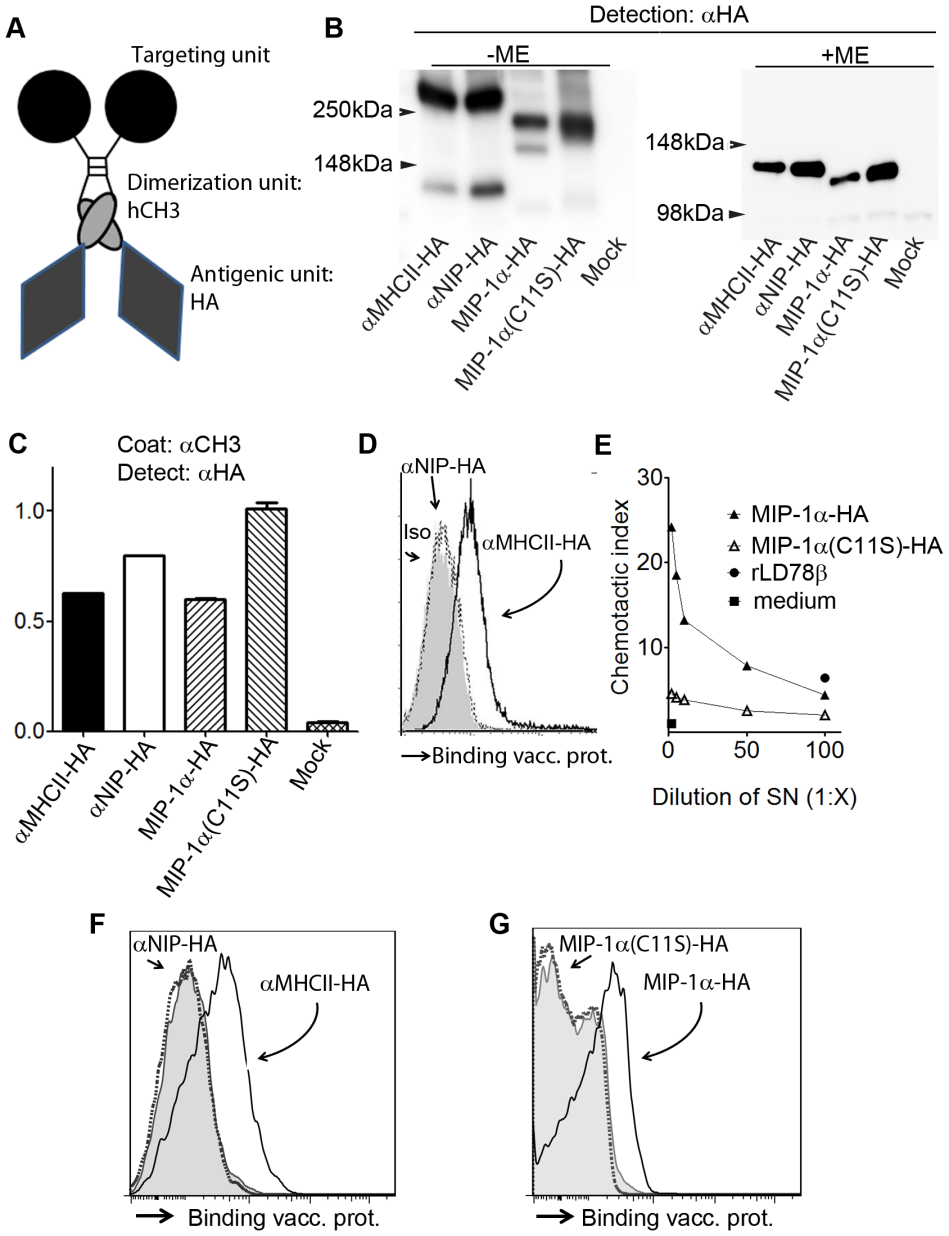
Characterization of fusion vaccine proteins. a) Schematic overview of homodimeric vaccine proteins. The fusion proteins consists of HA antigen connected to a targeting unit via a shortened Ig hinge and a dimerizing human γ3 CH3 domain and Ig hinge. As targeting units we used a scFv directed against the MHC class II molecule I-E^d^ (αMHCII-HA), or the mouse chemokine MIP-1α (MIP-1α-HA). For non-targeted controls, a scFv directed against the hapten NIP (αNIP-HA), or a mutated MIP-1α (MIP-1α(C11S)-HA), replaced functional targeting units. b) Supernatants of transfected 293E cells were examined by Western blotting with anti-HA mAb under reducing (-ME) or non-reducing (+ME) conditions. Vaccine proteins are indicated below lanes, and MW by arrows. c) Binding of vaccine proteins to anti-C_H_3 mAb in Sandwich ELISA, followed by detection with an anti-HA mAb. d) Binding of vaccine proteins to MHCII I-E^d^-transfected L cell fibroblasts. Vaccine proteins were detected by anti-HA mAb. e) Supernatants of 293E cells transfected with MIP-1α-HA or the mutated counterpart (C11S) were examined for chemotaxis. Recombinant human MIP-1α(rLD78β) was included as positive control. Chemotactic index is shown. f, g) Binding of vaccine proteins to CD11b^+^ BALB/c splenocytes.

Amino acids 18–541 from influenza HA (A/PR/8/34 (H1N1)) (PR8) were cloned into previously described plasmids that either encoded a scFv against mouse MHC class II (I-E^d^)[20] (αMHCII-HA)[27] or the chemokine MIP-1α[22] (MIP-1α-HA) as targeting units (Fig.1a). In non-targeted counterparts, the targeting units were replaced by either a scFv against the hapten NIP[20] (αNIP-HA) or a mutated version of MIP-1α abolishing chemotactic properties[22] [MIP-1α(C11S)-HA]. We also prepared a plasmid encoding only HA (aa 18–541) in order to evaluate the induced immune response in the absence of the bivalent fusion protein structures[27].

DNA plasmids were transfected into HEK293E cells for examinations of proper structure and function of the different vaccine fusion proteins. Western blotting of supernatants demonstrated bands with the predicted sizes (Fig.1b), whereas ELISAs confirmed secretion of vaccine fusion proteins (Fig.1c). The vaccine proteins were for the most part covalently dimerized, but low amounts of monomers were also found (Fig.1b). The αMHC class II targeting unit was proven functional by assessment of protein binding to MHCII I-E^d^-transfected L-cell fibroblasts (Fig.1d) and BALB/c CD11b^+^ splenocytes (Fig.1f)[27]. Intact functionality of the MIP-1α encoding vaccine was demonstrated in a chemotactic assay (Fig.1e) and by binding to BALB/c CD11b^+^ splenocytes (Fig.1g).

### Targeted DNA vaccination increases immune responses following intramuscular delivery

**Figure 2:**
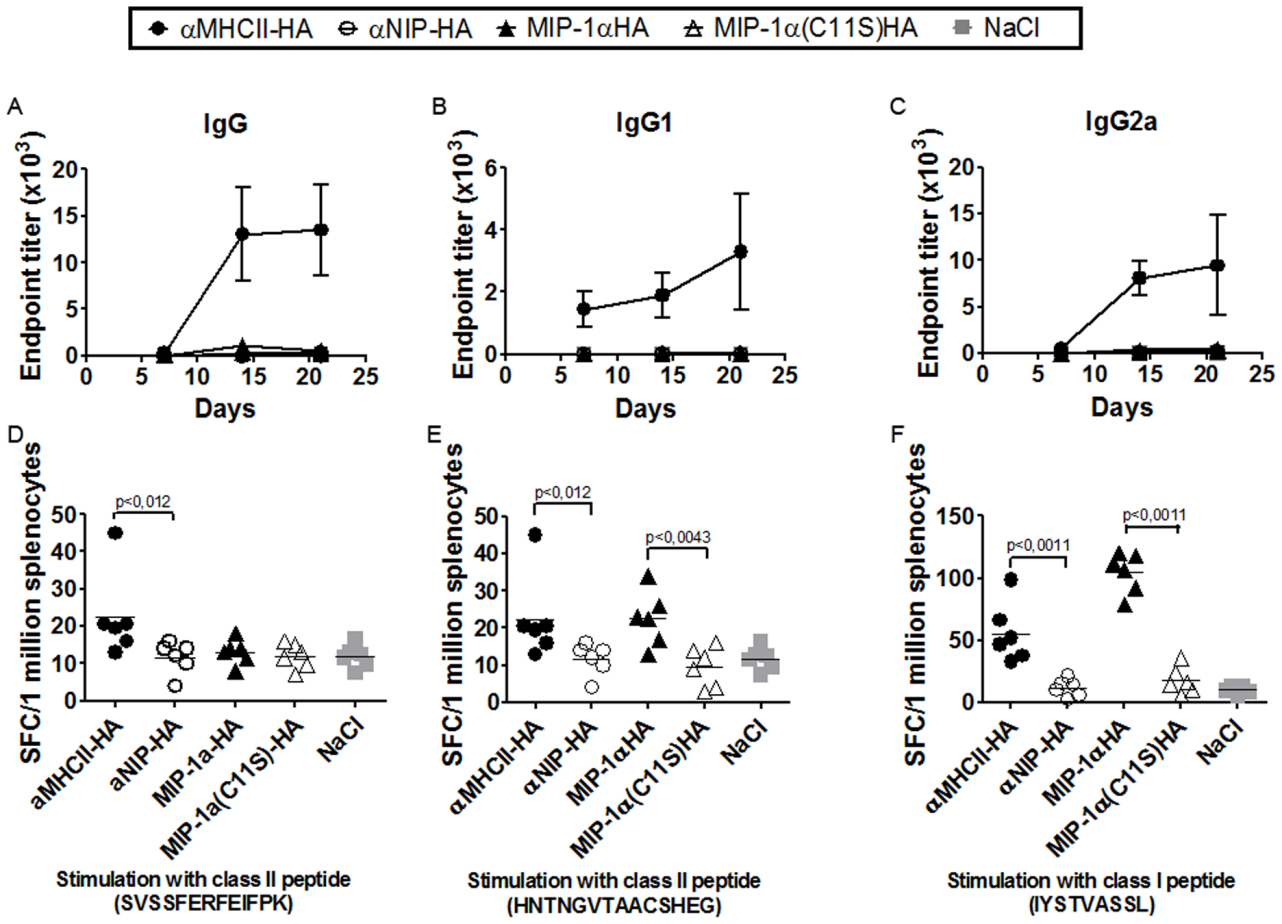
Targeted DNA fusion vaccines enhance immune responses after intramuscular delivery. Mice were vaccinated once i.m. with 25 µg DNA/electroporation (EP) as indicated (n = 6/group). (a-c) Serum samples were assayed for total IgG (a), IgG1 (b) and IgG2a (c) against PR8 in ELISA (mean+/-SEM). (d-f) Three weeks after vaccination, splenocytes were harvested and stimulated *in vitro* with either class II restricted HA peptides [d, (SVSSFERFEIFPK) or e, (HNTNGVTAACSHEG)], a class I restricted HA peptide [f, (IYSTVASSL)], or a control peptide (GYKDGNEYI). Frequencies of IFNγ-producing cells were evaluated by EliSpot. The control peptide did not elicit responses beyond that observed for NaCl. Horizontal lines indicate sample means.

BALB/c mice were vaccinated once by intramuscular (i.m.) injection of DNA vaccines immediately followed by electroporation to enhance DNA uptake. Sera obtained at day 7, 14 and 21 after vaccination with αMHCII-HA showed large increases in levels of total IgG, IgG1 and IgG2a in ELISA against PR8 (Fig.2a-c). By comparison, vaccinations with MIP-1α-HA and non-targeted controls induced only minor amounts of antibodies.

For assessment of T cell responses, spleen cells harvested at day 21 were stimulated with either MHC class II restricted HA peptides (SVSSFERFEIFPK or HNTNGVTAACSHEG), a class I restricted HA peptide (IYSTVASSL)[32]–[34], or a control peptide (GYKDGNEYI). EliSpot analysis demonstrated significantly increased frequencies of interferon gamma (IFNγ)-secreting cells after a single vaccination with the APC targeted vaccines, with MIP-1α-HA being particularly effective following stimulation with the class I peptide IYSTVASSL (p<0,0043 as compared to αMHCII-HA) (Fig.2d-f).

### Vaccination with αMHCII-HA increases humoral responses following intradermal delivery

Evaluation of DNA vaccines in preclinical models is often performed with i.m. delivery of DNA in combination with electroporation. However, intradermal (i.d) delivery may be clinically more tolerable since skin is easier accessible than muscle, and shorter needles are needed[35]. Furthermore, skin is rich in APCs, such as Lagerhans cells and dermal dendritic cells[36]. Therefore, we did in further experiments employ i.d. vaccination.

**Figure 3:**
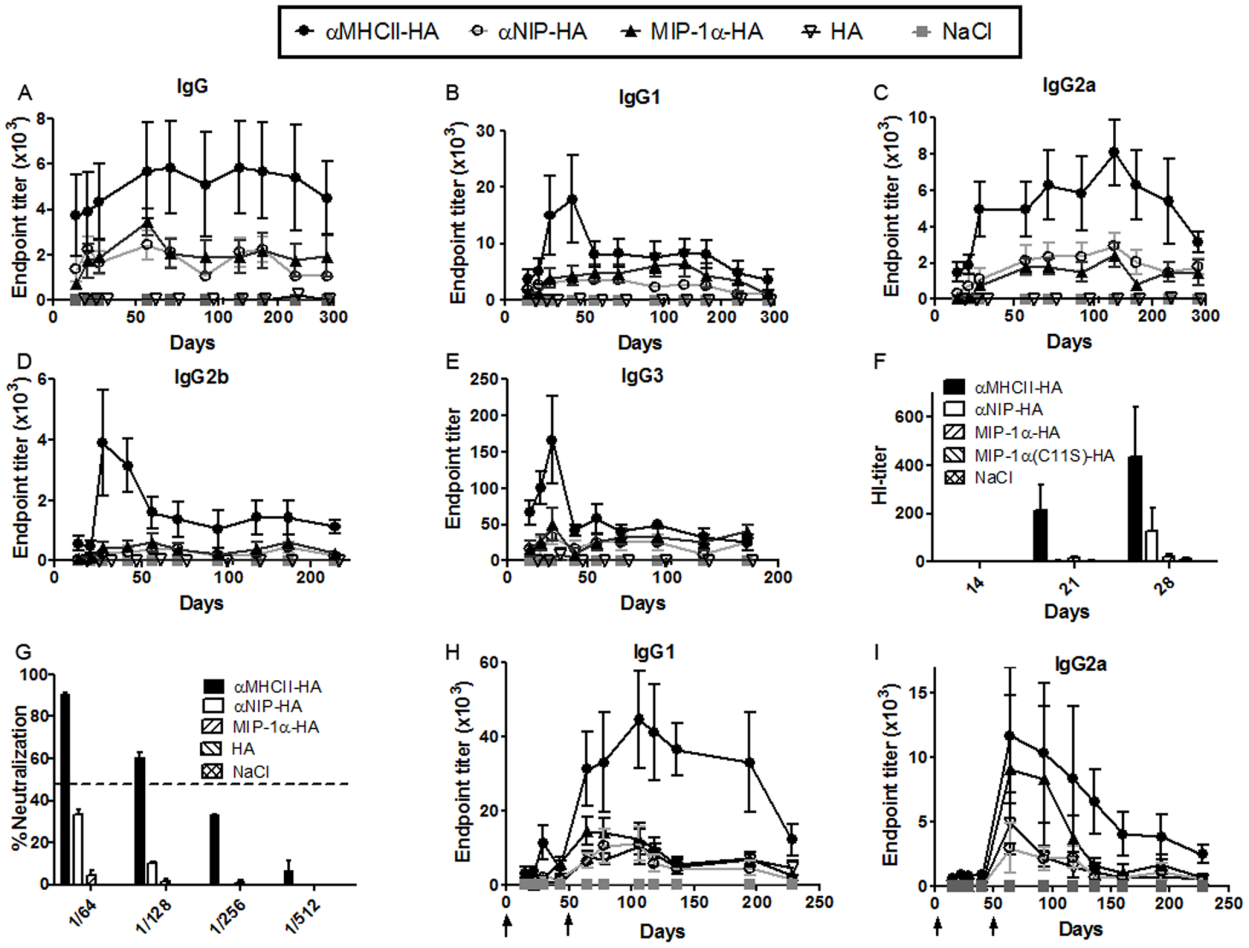
Antibodies in sera after intradermal DNA vaccination. (a-g) Mice were immunized once i.d. with 25 µg DNA/EP as indicated (n = 6/group), and assayed for total IgG (a), IgG1 (b), IgG2a (c), IgG2b (d) and IgG3 (e) against PR8 in ELISA (mean+/-SEM). f) Hemagglutination-inhibition (HI) titers (mean+/-SEM) in sera. g) Sera from day 14 were assayed in a micro neutralization assay (PR8 virus). Dotted line indicates threshold for positive neutralization (50%). (h,i) Mice were immunized twice i.d. at days 0 and 50 as indicated by arrows (↑), and sera assayed in ELISA against PR8 for induced IgG1 (h) and IgG2a (i) (mean +/- SEM).

BALB/c mice were vaccinated once i.d. with the DNA plasmids described above in combination with electroporation, and serum antibodies against PR8 were measured in ELISA. αMHCII-HA rapidly induced high and long-lasting titers of IgG1 and IgG2a, whereas the smaller increases of IgG2b and IgG3 declined to just above baseline within 50 days (Fig.3a-e). The increases in ELISA antibody titers following vaccination with αMHCII-HA were matched by increased hemagglutination inhibition (HI) and micro neutralizing titers (Fig.3f,g). In contrast, a single vaccination with MIP-1α-HA failed to increase antibody titers in ELISA beyond that observed for non-targeting controls, and hardly any antibodies were detected in the HI- and microneutralization assays. Immunization with HA alone failed to induce anti-HA antibodies in any of the assays.

To assess the effect of repeated immunizations, mice were vaccinated twice with a 50-days interval. Sera were collected at various timepoints and assayed against PR8 in ELISA. Results demonstrated that the boost with αMHCII-HA further enhanced both IgG1 and IgG2a titers (Fig.3h,i). By comparison, the boost with MIP-1α-HA failed to increase IgG1 levels beyond that observed for αNIP-HA (Fig.3h). In striking contrast, the MIP-1α-HA boost increased serum levels of IgG2a titers to levels comparable to that of αMHCII-HA from about day 70 to 120, after which a decline back to background levels was seen (Fig.3i). Repeated immunizations with HA alone induced antibody titers comparable to the non-targeted control αNIP-HA.

### Targeting of HA to either CCR1/3/5 or MHCII induces different T cell phenotypes

**Figure 4:**
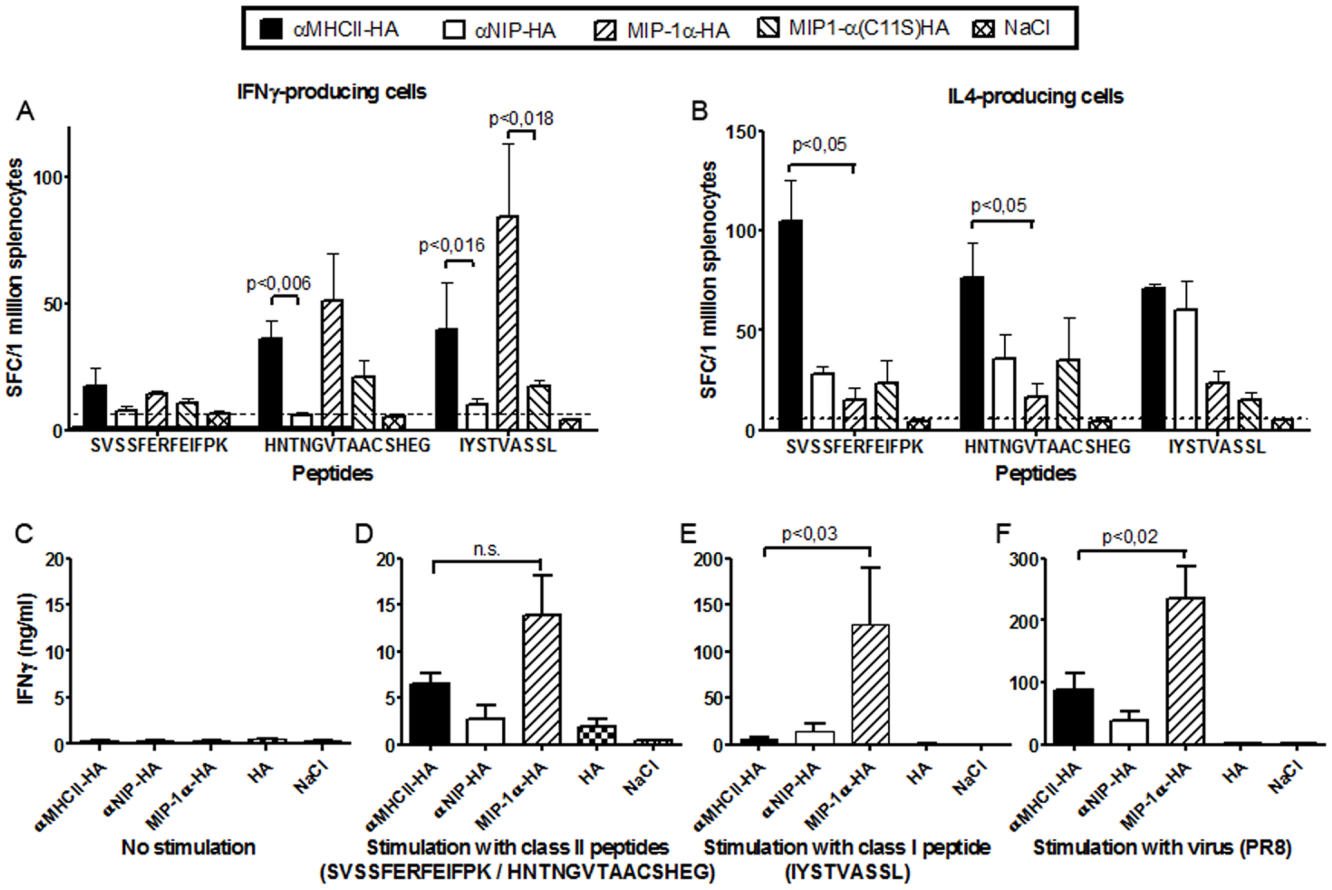
T cell activation following intradermal DNA vaccination. Mice were immunized once i.d. with 25 µg DNA/EP as indicated (n = 6/group). (a, b) Fourteen days after vaccination, splenocytes were harvested and analysed in EliSpot assays for IFNγ (a) or IL-4 (b) production following *in vitro* stimulation with class II restricted HA peptides (SVSSFERFEIFPK or HNTNGVTAACSHEG), a class I restricted HA peptide (IYSTVASSL), or a control peptide (GYKDGNEYI) (mean+/-SEM). The dotted lines indicate the highest responses found after stimulation with the control peptide. (c-f) Fifteen days after a single DNA/EP vaccination, splenocytes were stimulated *in vitro* with medium alone(c), a 1∶1 combination of two class II restricted HA peptides (SVSSFERFEIFPK and HNTNGVTAACSHEG)(d), a class I restricted HA peptide (IYSTVASSL)(e), or inactivated PR8 virus (f). Supernatants were analyzed in IFNγ-ELISA (mean+/- SEM). In d-f, MIP-1α-HA induced significant IFNγ-responses compared to either αNIP-HA, or HA, or NaCl (p<0.05).

Splenocytes harvested 14 days after one i.d. vaccination were stimulated *in vitro* with either class II restricted HA peptides (SVSSFERFEIFPK or HNTNGVTAACSHEG), a class I restricted HA peptide (IYSTVASSL), or a control peptide (GYKDGNEYI). EliSpot analysis of the relative amounts of cells secreting IFNγ showed that targeting of HA to either MHCII or CCR1/3/5 resulted in increased T cell activation as compared to the non-targeted controls (Fig.4a). However, IFNγ-secretion was particularly enhanced following vaccination with MIP-1α-HA, and especially after stimulation with the class I restricted peptide. In a separate experiment, splenocytes from vaccinated mice were stimulated *in vitro* with the above peptides and the levels of secreted cytokines assessed in ELISA (Fig.4c-f). This experiment confirmed a strong increase in IFNγ secretion following vaccination with MIP-1α-HA, as compared to αMHCII-HA and non-targeted controls.

An examination of the relative numbers of interleukin-4 (IL-4)-producing cells gave the opposite result. Thus, EliSpot analysis of splenocytes collected 14 days after vaccination and stimulated with either of the class II restricted HA peptides, demonstrated increased IL-4 production after vaccination with αMHCII-HA (p<0,05, compared to MIP-1α-HA) (Fig.4b). Vaccination with MIP-1α-HA did not elicit IL4-producing cells at all. Taken together, these results indicate that targeting with MIP-1α induces a Th1-like response, whereas targeting to MHC class II molecules predominantly induces a Th2-like response.

### Both CCR1,3,5- and MHCII-targeted vaccines protect against influenza

**Figure 5:**
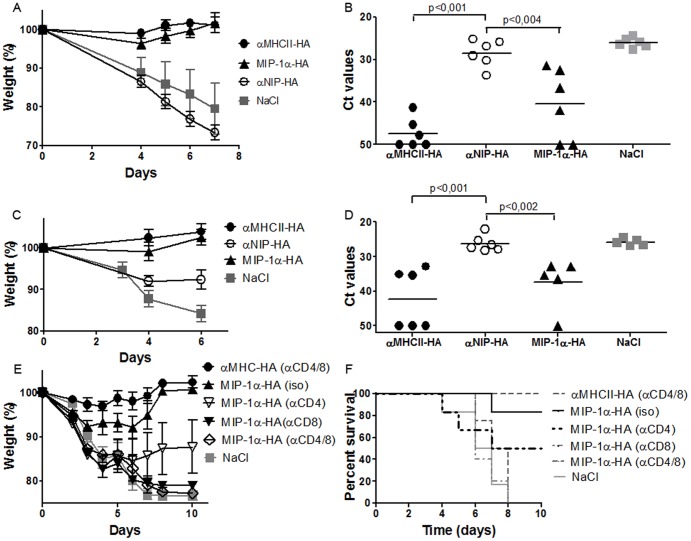
Targeted DNA vaccines protect mice against a lethal challenge with influenza virus. Mice were vaccinated once i.d. with 25 µg of the indicated DNA vaccines/EP. (a, b) Fourteen days after vaccination, mice were given a lethal challenge with influenza PR8. (a) Weight loss after challenge (mean weight+/-SEM). (b) Viral load in nasal washes from day 6. Ct-values for individual mice are shown. (c, d) Nine months after a single immunization, mice were given a lethal challenge with PR8 influenza virus and monitored for weight loss (c) and viral load (day 6) (d). Ct-values for individual mice are shown. (e, f) Mice immunized once with DNA/EP were from day 12 on injected with depleting antibodies against CD4, CD8, both, or isotype matched controls. On day 14, mice were challenged with a lethal dose of influenza PR8 virus, and monitored for weight loss (e) and survival (f).

BALB/c mice were vaccinated once i.d. with DNA/EP and challenged with influenza virus A/PR/8/34 (H1N1) (PR8) 14 days later. Mice vaccinated with αNIP-HA or NaCl rapidly lost weight, and had to be euthanized by day 7. In contrast, mice vaccinated with αMHCII-HA showed no weight loss or other signs of discomfort. Vaccination with MIP-1α-HA did not completely prevent weight loss, and a minor reduction in weight was observed between days 3 and 5 (Fig.5a). Viral load was examined by RT-PCR analysis of nasal washes collected from the infected animals (Fig.5b). Results from day 6 demonstrated that all mice receiving either αMHCII-HA or MIP-1α-HA had reduced viral titers as compared to αNIP-HA (p<0,002 and p<0,004, respectively). The reduction in viral load was more pronounced for αMHCII-HA, than for MIP-1α-HA.

In a separate experiment, mice were immunized once i.d. and challenged 9 months later with influenza. Again, vaccination with αMHCII-HA completely protected mice against weight loss, whereas mice vaccinated with MIP-1α-HA had a transient and moderate weight loss (Fig.5c). The long term protection after a single vaccination was confirmed by RT-PCR of viral load in nasal washes (Fig.5d).

### Targeting to MHC class II molecules induces antibody-mediated protection, whereas targeting to CCR1/3/5 induces cellular immunity

To examine T cell contribution to protection, mice were DNA vaccinated once with MIP-1α-HA and treated from day 12 and on with injections of depleting mAbs against CD8, CD4, or both. An additional group was treated with isotype matched mAbs. For comparison, a group of mice was vaccinated with αMHCII-HA and treated as above with depleting antibodies against both CD4 and CD8. All mice were challenged with influenza PR8 virus 14 days after vaccination, and monitored for weight loss. Depletion with mAbs against CD8 and CD4 had no effect on protection following vaccination with αMHCII-HA, suggesting that the large amounts of vaccine-induced HA-specific antibodies represent the main mechanism of protection. By contrast, T cell depletion abrogated the protection induced by vaccination with MIP-1α-HA. CD8^+^ T cells were absolutely required for protection while CD4^+^ T cells had a partial protective effect (Fig5e,f).

### Targeting of ovalbumin to MHC class II molecules increases antibody responses, whereas targeting to CCR1/3/5 increases T cell activation

**Figure 6:**
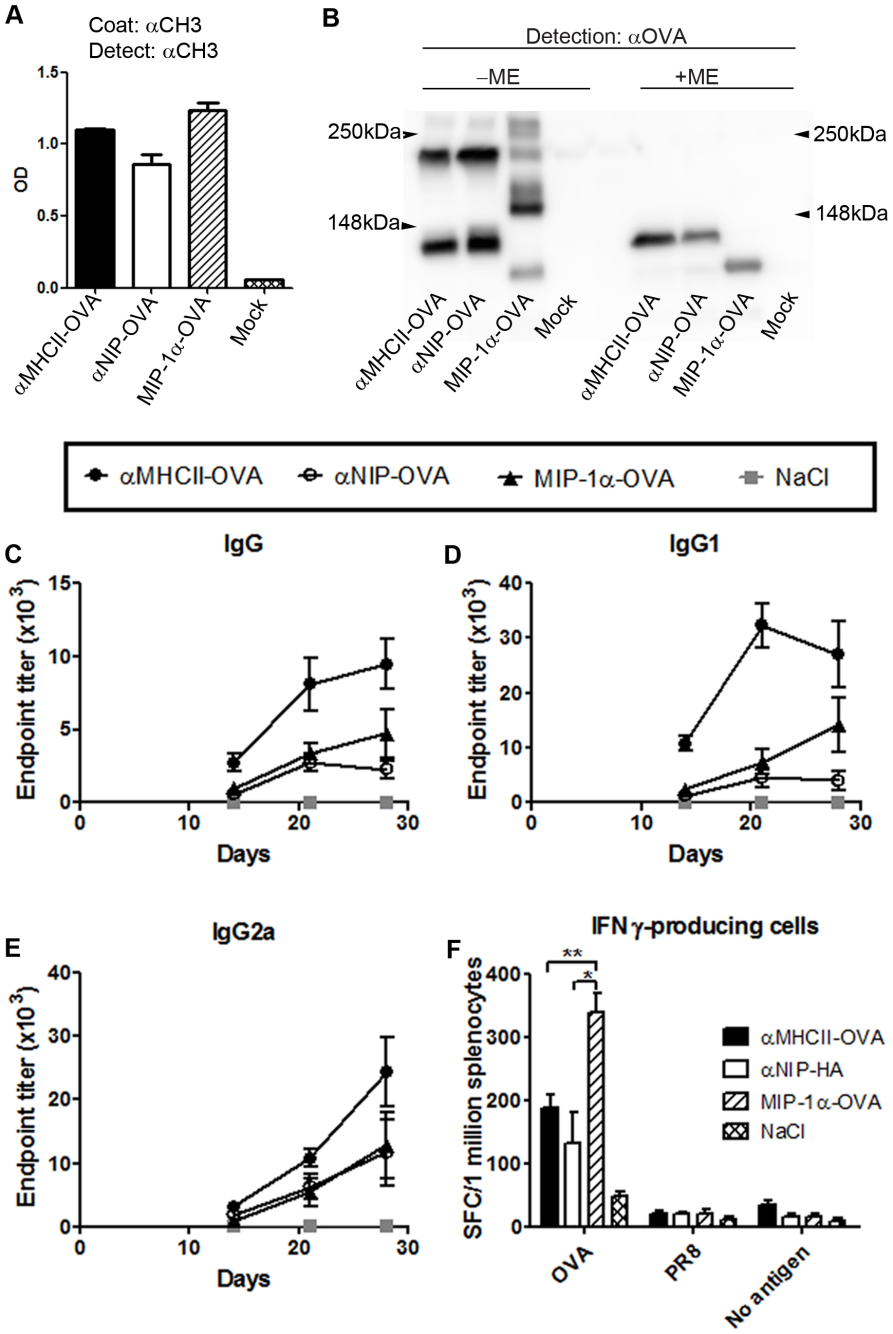
Antibody and T cell responses following vaccination with OVA targeted to MHC class II molecules or CCR1/3/5. (a,b) Supernatants of 293E cells transfected with the indicated plasmids were tested for secreted proteins in ELISA (a) and examined by Western blotting with anti-OVA mAb under reducing (+ME) or non-reducing (-ME) conditions (b). Vaccine proteins are indicated below lanes. (c-f) Mice were immunized once i.d. with 25 µg DNA/EP, as indicated. (c-e) Sera were assayed for total IgG (c), IgG1 (d) or IgG2a (e) against OVA. (f) Splenocytes collected at day 14 post immunization were stimulated *in vitro* with OVA protein or controls as indicated, and analyzed by an IFNγ EliSpot. *indicates p<0.008 and **p<0.002.

To test whether the above results could be extended to another antigen, HA was exchanged for ovalbumin (OVA) in the antigenic unit of the homodimeric vaccine constructs. Transfectants secreted vaccine proteins, but about half of these were monomers indicating inefficient covalent homodimerization (Fig.6a-b). BALB/c mice were immunized once i.d., and sera from different time points were analysed in ELISA for OVA-specific antibodies. Vaccination with αMHCII-OVA increased antibody responses as compared to MIP-1α-OVA and αNIP-OVA (Fig.6c-e). The increase was particularly evident for IgG1 (Fig.6d). T cell responses in vaccinated mice were examined by EliSpot, and demonstrated a significant increase in IFNγ-secretion following vaccination with MIP-1α-OVA as compared to αMHCII-OVA (p<0,002) or αNIP-OVA (p<0,008) (Fig.6f).

## Discussion

Efficient host responses against intracellular bacteria and viruses generally require Th1 cells and CD8^+^ T cells, whereas protection against extracellular pathogens requires antibodies and Th2 cells. It is therefore important to develop vaccines that can induce the particular immune response required to fend off a given pathogen. Previously, others have demonstrated that Th1/Th2 polarization of CD4^+^ T cells can be influenced by differences in vaccine particle size[37], vaccination with an antigen that has been conjugated to mannan under reducing or oxidative conditions[38], or co-delivery of antigens and cytokines in the form of DNA[39]. However, to our knowledge, the target-specific induction of different immune phenotypes has not been investigated. Herein, we demonstrate that vaccination with HA can be modified to preferentially activate Th1 associated cellular responses or antibodies and Th2-like T cells. Such polarization may be obtained by targeting of HA antigen to CCR1/3/5 and MHC class II molecules, respectively. The results were extended to a different antigen (OVA), indicating that the principle should be applicable to a variety of pathogens.

The observed polarizing effect may be caused by either (i) the particular APC surface molecule that was targeted or (ii) the particular APC that displayed the targeted surface molecules. As for the first mechanism, signaling through MHC class II molecules, induced by αMHCII-HA vaccine proteins, could somehow poise the APC for an ability to direct naive T cells towards Th2 polarization. Conversely, vaccination with MIP-1α-HA could induce signaling through CCR1,3,5 that would brace the APC for an ability to induce Th1 differentiation. As for the second mechanism, MHC class II molecules are displayed by dendritic cells, B cells and macrophages[40] whereas CCR1,3,5 are expressed on monocytes, macrophages, dendritic cells, lymphocytes, NK cells, eosinophils, basophils, platelets, neurons, microlial cells, fibroblasts and endothelial cells[41]. However, splenocyte stainings indicated that MIP-1α-HA preferentially bound CD11b^+^ cells (Fig.1g, and unpublished material), suggesting that monocytes and macrophages could be particularly important mediators of the Th1 dominance observed after CCR1,3,5 targeted vaccination. As for the second mechanism, different receptors on CD8^+^ DCs (such as Clec9A, DEC205 and Langerin) have been demonstrated to exhibit similar potentials for induction of Th1 and CD8^+^ immunity[42]. These results indicate that cross-presenting CD8^+^ DCs preferentially induce CD8^+^/Th1 responses regardless of what surface molecule is targeted on this type of APC. To assess whether the particular surface molecule is of relevance, or if the targeted cell subtype is indeed the determining factor, further investigations are required. Finally, it should be emphasized that the surface molecule targeted, and the cell type, together could influence the outcome in terms of polarization.

The APC-targeted fusion proteins were delivered in the form of DNA. We have previously shown that such vaccines are enhanced by two factors working in synergy: (i) an APC-targeted fusion protein encoded by the DNA and (ii) electroporation (EP) of the DNA injection site. EP increases transfection efficacy[43], [44] and production of secreted vaccine proteins[20]. Furthermore, EP has been reported to induce local inflammation, and secretion of Th1-associated cytokines[45], [46] at the site of injection. Despite this, targeting of fusion proteins MHC class II molecules induced a skewed Th2 response, indicating that the targeting effect is dominant over the EP effect for Th polarization. Furthermore, we here show only minor immune responses after vaccination with non-targeted controls in muscle, demonstrating that the vaccine-induced effect was dependent upon APC-targeting even in the presence of EP.

The polarized induction of dominant Th1 or Th2 immune responses after vaccination with MIP-1α-HA and αMHCII-HA, respectively, appeared to be independent of vaccination site, since a similar polarization was observed for both i.m. and i.d. DNA/EP vaccination. A striking difference between i.m. and i.d. vaccination, however, was the almost complete lack of immune responses in muscle following vaccination with non-targeted vaccines. This difference was particularly evident for antibody responses. The reason for the difference was not investigated, but may be related to a higher density of APC in skin as compared to muscle. The higher APC density in skin is likely to facilitate improved uptake and presentation of non-targeted vaccine proteins.

Apart from skewing of T cell responses, the targeted vaccines increased the magnitude of immune responses as compared to the non-targeted control versions, both in terms of antibody and T cell responses. Thus, a single i.d. vaccination with αMHCII-HA enhanced IgG titers as compared to controls. A boost further increased antibody levels after vaccination with αMHCII-HA, with IgG1 being particularly enhanced. For MIP-1α-HA, a boost vaccination was needed to induce high IgG2a titers. As concerns T cell responses, a single vaccination with αMHCII-HA resulted in a significant increase of IL-4 secreting Th2 cells as compared to the non targeted control. Similarly, one vaccination with MIP-1α-HA increased Th1 responses as compared to non targeted controls. These results are in general agreement with the finding that targeting of antigens to APC is known to enhance the immunogenicity of subunit vaccines[10]–[27].

Antibodies represent a well-established correlate of protection for influenza[47], but current influenza vaccines need to be reformulated each year due to antigenic drift rendering last years' antibodies partly or completely ineffective against the new strain. The complete absence of disease after influenza virus challenge of αMHCII-HA vaccinated mice indicates that sterilizing Ab-mediated immunity was induced. This is consistent also with strongly reduced viral loads in these animals. Moreover, the high amounts of neutralizing Abs in the αMHCII-HA-vaccinated mice, and the fact that depletion of both CD4^+^ and CD8^+^ T cells did not abrogate protection, confirmed that αMHCII-HA induced Ab-mediated protection.

Following ligation, the chemokine receptor CCR5 is phosphorylated and endocytosed via clathrin-coated vesicles[48]. In agreement with receptor-mediated endocytosis, chemokine fusion proteins targeting chemokine receptors have been demonstrated to stimulate efficient vaccine uptake and presentation of antigenic peptides both in the context of MHC class I[21] and class II molecules[18]. The ability of an exogenous vaccine to induce CD8^+^ T cell responses, called cross-presentation, is a highly desirable trait that is important for eradication of virus-infected cells and tumor cells. The potency of chemokine receptor targeting in induction of CD8^+^ T cell responses is supported by previous studies demonstrating that MIP-1α-idiotypic tumor antigen fusion proteins are highly efficient at preventing cancer in mice[17], [18], [21], [22].

T cells can also protect against influenza virus[49], and both CD4^+^ and CD8^+^ T cells can independently confer protection[50]–[52]. A single vaccination with MIP-1α-HA induced strong HA-specific Th1 and CD8^+^ responses, where CD8^+^ T cells and CD4^+^ T cells contributed to protection. The T cell responses were presumably augmented by simultaneous MHC class I and II presentation of antigenic HA peptides on targeted APCs[53]. MIP-1α-HA did not prevent the establishment of influenza infection, as can be inferred from the slight weight decrease observed after viral challenge, but rather induced cytotoxic T cells that cleared already infected cells[54]. Furthermore, a single vaccination conferred protection against influenza that lasted at least 9 months, possibly indicating an initial CD4^+^ T cell contribution that could have facilitated the development of protective memory CD8^+^ T cells[55], [56]. For influenza vaccination, the induction of strong T cell responses hold promise for development of novel vaccines that may confer cross protection against a wider range of influenza strains.
